# Local and latitudinal variation in abundance: the mechanisms shaping the distribution of an ecosystem engineer

**DOI:** 10.7717/peerj.100

**Published:** 2013-07-09

**Authors:** Gregory M. Crutsinger, Angélica L. Gonzalez, Kerri M. Crawford, Nathan J. Sanders

**Affiliations:** 1 Department of Zoology, University of British Columbia, Vancouver, Canada; 2 Department of Biology, Washington University in St. Louis, St. Louis, MO, USA; 3 Department of Ecology and Evolutionary Biology, University of Tennessee, Knoxville, TN, USA

**Keywords:** Community ecology, Biogeography, Soil nutrients, Plant-insect interactions, Genetic variation, Solidago altissima, Ecosystem engineer, Latitudinal gradient

## Abstract

Ecological processes that determine the abundance of species within ecological communities vary across space and time. These scale-dependent processes are especially important when they affect key members of a community, such as ecosystem engineers that create shelter and food resources for other species. Yet, few studies have examined the suite of processes that shape the abundance of ecosystem engineers. Here, we evaluated the relative influence of temporal variation, local processes, and latitude on the abundance of an engineering insect—a rosette-galling midge, *Rhopalomyia solidaginis* (Diptera: Cecidomyiidae). Over a period of 3–5 years, we studied the density and size of galls across a suite of local experiments that manipulated genetic variation, soil nutrient availability, and the removal of other insects from the host plant, *Solidago altissima* (tall goldenrod). We also surveyed gall density within a single growing season across a 2,300 km latitudinal transect of goldenrod populations in the eastern United States. At the local scale, we found that host-plant genotypic variation was the best predictor of rosette gall density and size within a single year. We found that the removal of other insect herbivores resulted in an increase in gall density and size. The amendment of soil nutrients for four years had no effect on gall density, but galls were smaller in carbon-added plots compared to control and nitrogen additions. Finally, we observed that gall density varied several fold across years. At the biogeographic scale, we observed that the density of rosette gallers peaked at mid-latitudes. Using meta-analytic approaches, we found that the effect size of time, followed by host-plant genetic variation and latitude were the best predictors of gall density. Taken together, our study provides a unique comparison of multiple factors across different spatial and temporal scales that govern engineering insect herbivore density.

## Introduction

Understanding patterns in the abundance and the distribution of species across space and time is one of the core issues in ecology (Brown et al., 1995; Holt & Keitt, 2005). This is particularly the case for ecosystem engineers, as these species have cascading effects on the abundance of other members in a community by providing shelter from the physical environment, protection from enemies, or increased availability of food resources (Jones et al., 1994; Wright & Jones, 2006). For example, ecosystem-engineering insects, such as leaf tiers or leaf rollers, stem borers, and gall makers can create shelters for a suite of colonizing species that secondarily use these microhabitats (Lill & Marquis, 2003; Marquis & Lill, 2007; Calderón-Cortés et al., 2011; Crawford et al., 2007; Crutsinger et al., 2009; Vieira & Romero, in press).

At the local scale, a variety of processes might influence engineering insects, and, in turn, their effects on associated communities. As many engineering insect species are herbivores, variation in abundance and quality of their host plants may predict susceptibility to attack. For example, Marquis & Lill (2010) studied leaf-tying caterpillars on oak trees and found that the community composition of leaf-tying caterpillars and the secondary-users colonizing the ties varied among different oak species, which corresponded with leaf nutrients and defensive compounds. Yet, many host plants can also vary considerably within species in their susceptibility to insect herbivores. For instance, genetic variation within host-plant species can have strong effects on the population dynamics of associated herbivore species (Egan & Ott, 2007; Tack & Roslin, 2011; Underwood, 2012).

Along with host-plant genetic variation, the environment of the host plants could influence engineering insects, and, in turn, subsequently affect associated species. For example, variation in the abiotic environment, such as soil nutrient availability, may indirectly affect engineering insects by modifying host-plant quality (e.g., C:N ratios) or productivity (Stamp, 2003; Schade et al., 2003). Additionally, the biotic environment, such as the presence of other insect herbivores, could also affect subsequent colonization preferences and performance of engineering insects. For example, Van Zandt & Agrawal (2004) showed that damage by an early-season stem-feeding weevil reduced growth of monarch caterpillars and leaf beetle larvae on common milkweed (*Asclepias syriaca*). Taken together, understanding the influence of both the biotic and abiotic environment might be crucial to predicting engineering insect abundance at local scales.

At biogeographic scales, many taxa vary systematically with latitude (Hillebrand, 2004; Mittelbach et al., 2007; Field et al., 2009). Such variation is largely driven by changes in biotic, abiotic, historical and stochastic processes across latitudinal gradients (Gaston & Blackburn, 2000; Schemske et al., 2009). Hence, populations of insect engineers could vary with latitude and the corresponding changes of abiotic conditions, quality or quantity of host plants, or the abundance of natural enemies (Marczak et al., 2011). Indeed, the intensity of herbivory, in particular, has been shown to decline with latitude in a number of different systems (Pennings et al., 2009; Garibaldi et al., 2011; Salazar & Marquis, 2012), though little is known about the role of biogeographic processes for predicting the abundance of engineering insects.

Finally, engineering insect populations can fluctuate widely from one growing season to the next or across several years (Myers, 1990), which, in turn, could modify their influence within a community. For example, the overall abundance of engineering insects varies seasonally, likely resulting from such factors as host plant phenology (Forkner et al., 2008), abiotic conditions, or disturbance from one year to the next (McCrea & Abrahamson, 1987). In addition, recent evidence suggests the community-level impacts of engineering can also vary seasonally (Vieira & Romero, in press). While some studies have followed engineering insects over time (Marquis & Lill, 2007; Crawford et al., 2007; Crutsinger et al., 2009; Calderón-Cortés et al., 2011), the effect of time is rarely an explicit consideration in any study (but see Vieira & Romero, in press).

**Figure 1:**
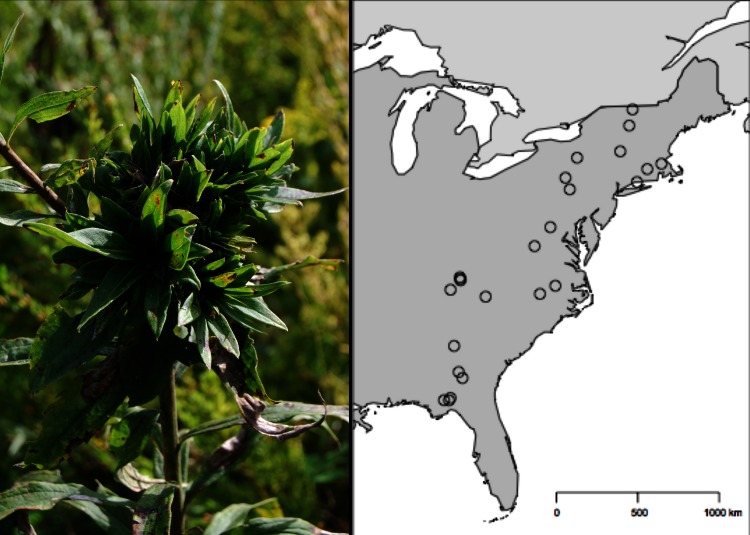
Geographic distribution of the 24 *Solidago altissima* populations surveyed for *Rhopalomyia solidaginis* across the eastern United States. The sites covered a region that represents a 14° variation in latitude along a 2,300 km transect, from the Florida Panhandle to Northern Vermont.

Here, we focus on the rosette-galling midge, *Rhopalomyia solidaginis* (Diptera: Cecidomyiidae), which is associated with tall goldenrod (*Solidago altissima*). This midge (hereafter referred to as ‘rosette galler’) initiates galls through oviposition in the apical meristem of *S. altissima*, preventing further elongation of the stem and creating a conspicuous rosette of leaves typically at the tip of the plant and on lateral buds (Fig. 1) (Raman & Abrahamson, 1995; Wise et al., 2006). The leaf rosettes created by the galler can be considered an engineered ecosystem as they are then secondarily colonized by ∼75 different secondary-user species. The presence of a rosette gall results in a doubling of the richness and abundance on a *S. altissima* stem and significantly alters the arthropod community composition (Crawford et al., 2007; Crutsinger et al., 2009). Despite the influence of the rosette galler on numerous other taxa and the host plant (Crawford et al., 2007), there is a surprising dearth of understanding about the factors governing its abundance at different spatial scales. In this study, we took advantage of several separate field experiments manipulating the identity of *S. altissima* genotypes, the availability of soil nutrients, and the presence of other insect herbivores at a single local field site in East Tennessee. We also combined the experimental work with a biogeographic survey of rosette gallers along a 14°(∼2300 km) latitudinal gradient in the eastern United States. The overarching goal of this study was to compare the individual contributions of local and biogeographic processes on the density of rosette gallers. Specifically, we ask the following questions: (1) At local scales, what are the relative effects of plant genetic variation, soil nutrients, the presence of other insects, and time on the density of rosette gallers? (2) Is there systematic variation in the density of rosette gallers at latitudinal scales? (3) What are the relative effect sizes of local and biogeographic factors, as well as time on rosette galler density?

## Methods

### Study system

The focal host plant of the rosette galler is *Solidago altissima* (tall goldenrod), a rhizomatous, perennial forb that dominates meadows, abandoned agricultural fields, and roadsides throughout eastern North America (Semple & Cook, 2006). Local populations of *S. altissima* vary in size from a single ramet to thousands of ramets (Maddox et al., 1989), and previous work in this system has indicated considerable genetic variation in susceptibility of *S. altissima* to rosette gallers (Crawford et al., 2007; Crutsinger et al., 2009). We conducted the local-scale experiments at Freels Bend, which is part of the Oak Ridge National Laboratory National Environmental Research Park near Oak Ridge, Tennessee. The site consists of numerous old fields that are mowed annually to prevent succession. Soils at the sites are characterized as Typic Hapludult with a silty clay loam texture. Mean monthly temperatures range from approximately 3°C in the winter to 31°C in the summer and mean rainfall is 1322 mm. The old fields are typical of the region, and *S. altissima* is one of the most common species at the site (Souza, Weltzin & Sanders, 2011).

#### Local factors: genetic variation

To estimate how *S. altissima* genetic variation influences rosette galler density, we surveyed a common garden experiment established in 2005 consisting of 1- m^2^ plots initially planted with 12 ramets of one of 21 distinct *S. altissima* genotypes (genotypic monocultures). There were two replicate plots for each genotype. Ramets were collected from local *S. altissima* patches growing in fields surrounding the study site, and we identified each ramet as a unique genotype by means of amplified fragment length polymorphisms (AFLPs). Genotypic monocultures were arranged randomly within the common garden and allowed to spread clonally within experimental plots. Though all 1- m^2^ plots contained 12 individuals in 2005, genotypes varied from 80–190 ramets the following years. For further details on the study site and common garden experiment see Crutsinger et al. (2006) and Crawford et al. (2007). In 2005, 2006 and 2007, we estimated rosette galler density by counting the number *S. altissima* stems and the number of galls in each plot. In 2006, we also determined whether gall size varied among genotypes by measuring the biomass of five galls from each plot. Gall size has been shown to be positively correlated with the diversity of secondary-users of these microhabitats (Crutsinger et al., 2009).

#### Local factors: soil nutrient availability and herbivore competitors

A separate experiment was established at Freels Bend in April 2004, ∼500 m from the genotype common garden, that manipulated soil nutrient availability and the presence of insects. Seventy-two plots (3 × 3 m, including a 0.5-m buffer around each plot) were established within an existing old-field community dominated by *S. altissima*, with 2-m spacing between plots. In the first 2 years of the study, propagule supply of an invasive plant species, *Lespedeza cuneata*, was also manipulated in 36 of the original 72 plots. However, the addition of *L. cuneata* had no influence on rosette gallers in any of our analyses, so *L. cuneata* was dropped as a factor in our models.

In a fully crossed, completely randomized plot design, we manipulated soil nitrogen (N) and the abundance of insects in randomly assigned plots. Our soil N manipulations consisted of (1) adding N (applied as urea fertilizer, at a rate of 20 g m^2^ year^-1^), (2) adding carbon (C) (applied as sucrose at a rate of 167 g m^2^ year^-1^) and (3) unmanipulated control plots. Rates of N addition were similar to other studies addressing the role of N fertilization on dynamics in grasslands and old fields (McLendon & Redente, 1991; Larson & Sieman, 1998). The addition of C in the form of sucrose provides microbial communities with a surplus source of labile C ultimately leading to N immobilization (Wang et al., 2004; Craine et al., 2007). In 2005, one year after the first application of the nutrient treatments, soil N availability (NO3-N + NH4-N) was two times greater in the N addition plots and 5 times lower in the N reduction plots than in the control plots (Sanders et al., 2007). Throughout the experiment, nitrogen availability in the nitrogen addition plots was consistently higher than either the control or sucrose addition plots. For further details on this experiment see Sanders et al. (2007).

The abundance of potential insect competitors and predators of rosette gallers was also manipulated at two levels: (1) unmanipulated controls in which insects were present and (2) the reduction of insects using a permethrin insecticide (Hi-Yield Kill-A-Bug; Voluntary Purchasing Group, Bonham, TX, USA) applied with a backpack sprayer at a rate of 0.23 L m^2^ every 2–3 weeks during the growing season. The use of pyrethroid-based insecticides effectively reduced insect abundance, as in other studies (Root, 1996; Schmitz, 2006), but appeared to have no negative influence on the abundance of rosette gallers. In fact, gall densities appeared to be higher in insecticide is added (see results and discussion). At the beginning of the experiment in 2004, insecticide was added in May after gall initiation had occurred. Therefore, 2004 gall densities can be considered as pre-treatment densities. Overall insect abundance was on average 4-fold lower in the insect reduced plots relative to the control plots (Sanders et al., 2007). Importantly, external feeding insect herbivores were by far the most abundant trophic group in control plots and their presence has been shown to negatively affect aboveground biomass of some plant species in the experiment (Sanders et al., 2007; Blue et al., 2011).

To estimate rosette galler density in this experiment, we haphazardly placed a 1- m^2^ quadrat within the 3 × 3 m experimental plots and counted the number of *S. altissima* stems and rosette gallers in August of each year from 2004 (pre-experimental initiation) to 2008. In 2006, we also measured the average size of rosette galls as a measure of habitat availability to secondary-user species. Galls (consisting of both leaves and chambers) were clipped at their base, oven dried for 48 h at 60°C and weight to the nearest 0.01 g.

#### Biogeographic variation in the density of rosette gallers

To estimate variation in rosette galler density at larger spatial scales, we sampled populations of *S. altissima* at 24 sites from the Florida Panhandle to northern Vermont (Fig. 1) from July–August of 2006, well after spring gall initiation when rosettes are clearly visible. Old-field ecosystem sites were selected haphazardly with two conditions: they had to be relatively free of recent disturbance (e.g., cattle grazing) and they had to contain at least several extensive patches (i.e., 10–30 m in length) of *S. altissima*. Within each site, we established a single 30-m transect with 10 1-m^2^ plots spaced 3 m apart. In each plot, we counted the number of *S. altissima* stems and visually surveyed each stem for rosette galls. We did not collect data on gall size in this survey.

### Statistical analyses

Rosette galler density was standardized across all local experiments and the latitudinal survey as ‘gall loads’ (Root, 1996). Gall loads were estimated as the number of rosette gallers/no. of *S. altissima* stems per m^2^, or the percent of stems galled (though occasionally stems contained multiple galls). We used repeated-measures ANOVA to determine whether gall load varied among the 21 *S. altissima* genotypes from 2005 to 2007 and a one-way ANOVA to examine whether gall size differed among genotypes in 2006 only. We also used repeated-measures ANOVA to analyze the main and interactive effects of nutrient manipulations and insect herbivory on rosette gall loads. We followed these analyses with separate two-way ANOVAs to examine responses within individual years. We calculated the percent variation (% Var) explained by all main effects using (SD/mean) × 100%. We used a two-way ANOVA to examine the effects of soil nutrients and insects on gall size in 2006.

To estimate the rosette gall loads across the latitudinal gradient, we fitted general linear models to assess the relationship between gall loads and latitude, longitude, and elevation. Ultimately, a simple polynomial regression model was the best fit for rosette gall loads and latitude, controlling for correlations with longitude (*r*^2^ = 0.79, *P* < 0.0001).

Finally, we compared relative explanatory power of the different factors (local, biogeography, time) on rosette galler loads by calculating the standard effect sizes. Effect size is a general term for the parameter used to measure the effect of a treatment or a variable within each study (Hillebrand, 2004). Here we used the *r*^2^ in the regression between rosette gall density and latitude as a measure of the effect size of latitude on rosette gall abundance, and the generalized eta-square (${\eta }_{G}^{2}$) for repeated-measures ANOVA (Olejnik & Algina, 2003) to estimate the effect sizes of genotype, nutrients, and insects. The η^2^ is a measure of association and hence can be interpreted in the same way as *r*^2^ (Bakeman, 2005). We also estimated the generalized eta-square (η^2^) for one-way ANOVA (Keppel & Wickens, 2004). We computed η^2^ for each factor (e.g., genotype, nutrients and insects), and year independently. We report the effect size results of the η^2^ for one-way ANOVAs, instead of using the ${\eta }_{G}^{2}$ for repeated-measures ANOVA (Olejnik & Algina, 2003) to show the proportion of variance explained per each factor per year. Results show the mean weighted effect sizes and 95% bootstrapped CIs. Cohen (1988) suggests a rule of thumb for characterizing effect sizes; ‘small’ effect = 0.10, a ‘medium’ effect = 0.30, and a ‘large’ effect = 0.50. All statistical analyses were performed with the statistical package R ver. 2.15.1 (R Development Core Term 2012).

## Results

**Figure 2:**
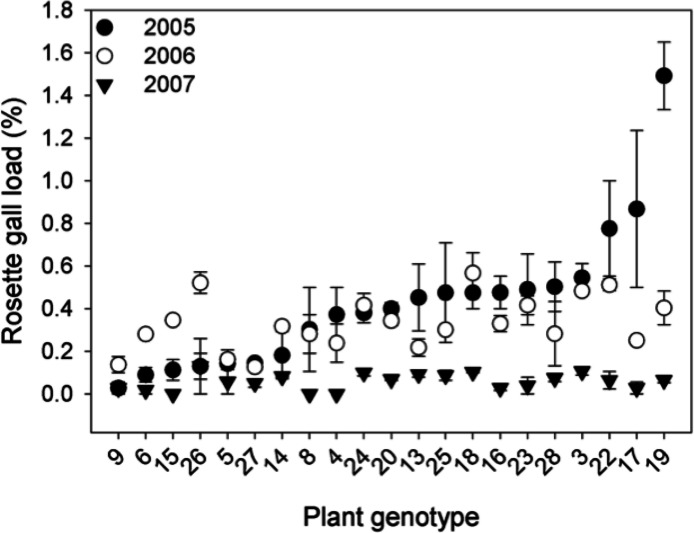
Rosette galler, *Rhopalomyia solidaginis*, density associated with 21 unique genotypes of *Solidago altissima* grown in a common garden, in 2005 (closed circles), 2006 (open circles) and 2007 (closed triangles).

**Figure 3:**
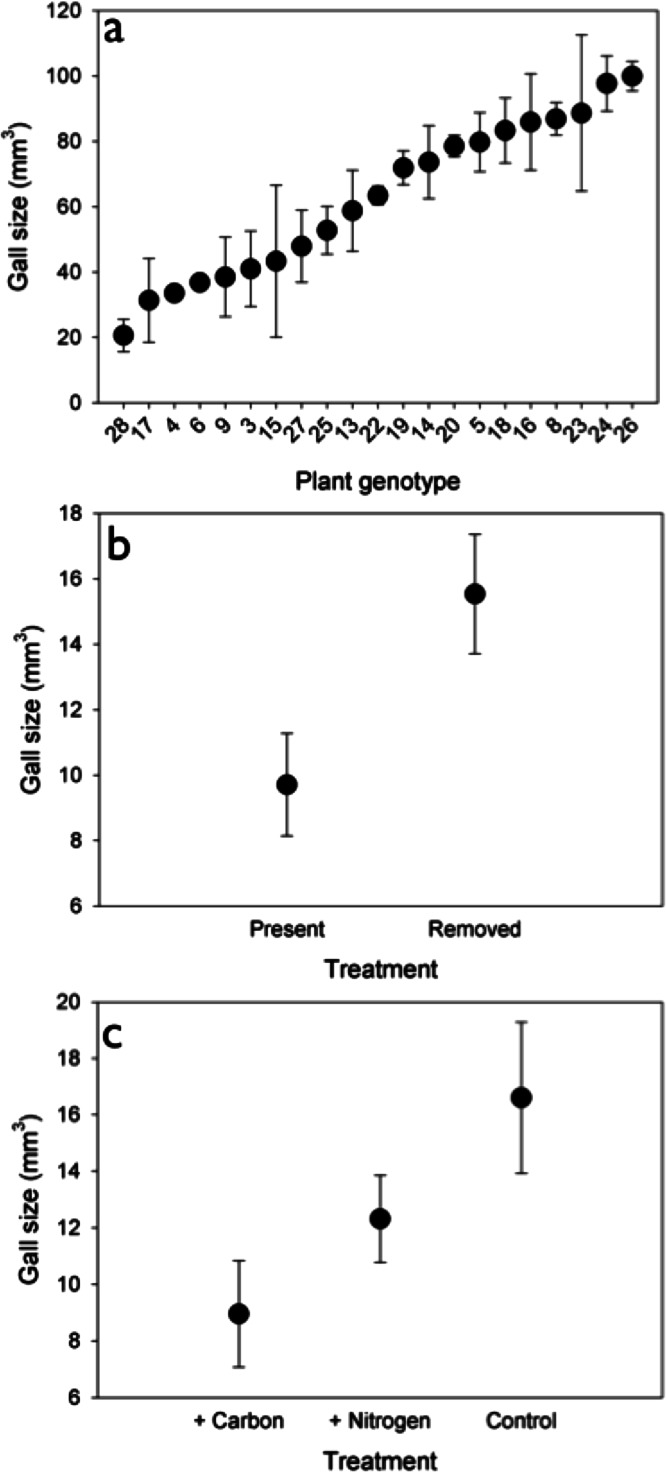
Size (mm^3^) of *Rhopalomyia solidaginis* rosettes associated with *Solidago altissima* plants in different local experiments, including (A) 21 unique genotypes of *S. altissima* grown in a common garden, (B) insect reduction through the application of insecticide, and (C) nutrient additions including N (supplied as urea fertilizer, at a rate of 10 g/m^2^), carbon (C; applied as sucrose at a rate of 167 g C/m^2^), and an unamended control. Circles represent the mean ( ± SEM).

*Genetic variation.* The density of rosette gallers varied significantly among *S. altissima* genotypes and years. Gall density varied by ∼16-fold, 4-fold, and 13-fold among the 21 genotypes in 2005, 2006, and 2007 respectively and plant genotype accounted for 67 to 83% of the total variation in rosette galler density across all years of the experiment (Fig. 2). Gall size also varied by 42% among genotypes (*F*_20,21_ = 4.70, *P* < 0.001) (Fig. 3A). Gall density varied by up to 8-fold between years, with densities of about one gall for every 2 *S. altissima* stems in 2005, every 3 stems in 2006, and every 17 stems in 2007. These results are likely driven by the rapid increase of stems in the common garden over time. Moreover, there was a significant interaction between *S. altissima* genotype × year (Table 1).

**Table 1:** Results of the repeated-measures ANOVA analyses for the effects of *Solidago altissima* genetic variation, soil nutrients, and insects on the density of *Rhopalomyia solidaginis* (rosette galler) in a common garden experiment.

Source of variation	*d* *f*	*F*	*P*
Genotype	20, 21	6.161	<**0.0001**
Year	2, 42	130.738	<**0.001**
Genotype × Year	40, 42	2.954	<**0.001**
Nutrients	2, 30	0.145	0.251
Insects	1, 30	5.408	<**0.05**
Nutrients × Insects	2, 30	0.129	0.879
Year	4, 120	42.529	<**0.0001**
Nutrients × Year	8, 120	0.600	0.776
Insects × Year	4, 120	3.233	<**0.05**
Nutrients × Insects × Year	8, 120	0.380	0.929

*Soil nutrient availability and Herbivore competitors.* We observed no effect of soil nutrient manipulation (either the addition of N or C) on the density of rosette gallers (Table 1). We found that gall density differed between insect treatments (Table 1), and density was 20% higher when insecticide was applied compared to control plots where insecticide was not applied. We did not observe any significant interactions between nutrients × insects or nutrients × year on gall density (Table 1). However, we found an overall effect of time: gall densities in 2007 and 2008 were lower than 2004, 2005 and 2006. We also found a significant interactive effect of insects × year on rosette gall density (Table 1). This interaction was driven by 2004 when spring gall initiation could potentially have occurred prior to the establishment of the experimental treatments.

Although there was no effect of nutrients on gall density, we did find an effect on gall size (*F*_2,30_ = 3.789, *P* < 0.05). Galls were 46% and 27% larger under control and N addition than under C addition (Fig. 3B). Insects also had a significant effect on gall size (*F*_1,30_ = 6.583, *P* < 0.05), such that galls were 40% larger when insects were removed (Fig. 3C) than when they were present.

**Figure 4:**
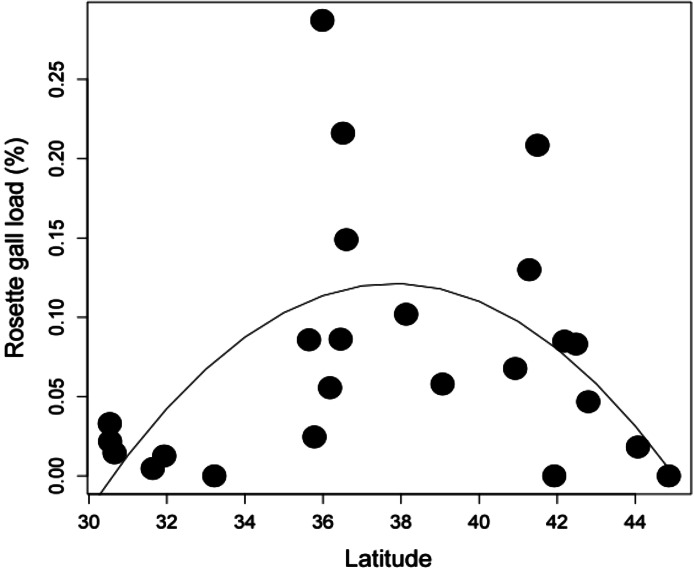
The relationship between *Rhopalomyia* galler density associated with *Solidago altissima* and latitude (*r*^2^ = 0.504, *P* < 0.05).

*Latitudinal gradient.* At the biogeographic scale, rosette galler density peaked at intermediate latitudes (Fig. 4) (*r*^2^ = 0.504, *P* < 0.05). This hump-shaped pattern remains consistent when the three mid-latitude sites containing the highest abundance were removed from the dataset.

**Figure 5:**
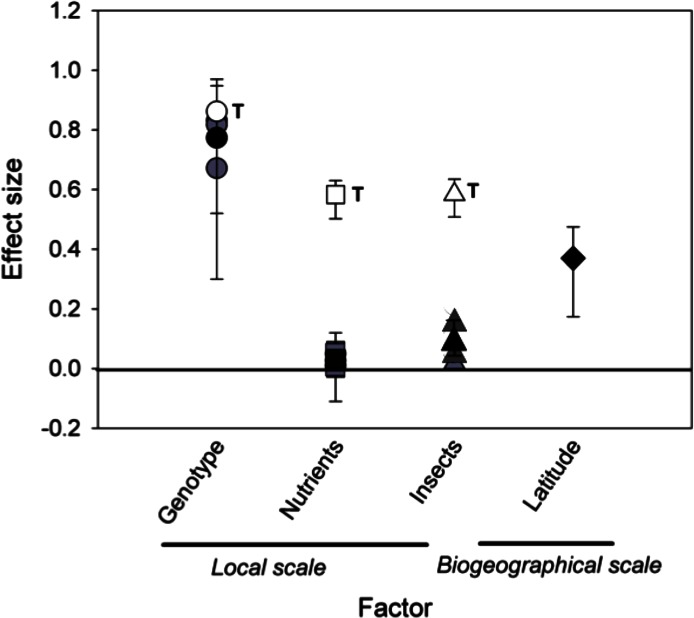
Mean effect sizes of local and biogeographical factors on rosette galler density. Black symbols show the mean effect sizes (eta squared (η^2^)) ± 95% of bootstrapped CIs. Grey symbols show yearly effect sizes for local scale factors, including *Solidago altissima* genotype (circles), nutrient additions (squares), and insect removal (triangles). White symbols show the effect size of time within each local treatment, also indicated by the letter “T”. The effect size of latitude is shown as a filled diamond. Confidence intervals that do not intercept zero indicate a significant effect of the respective factor based on *P* < 0.05.

*Comparing effect sizes.* When we compared the standard effect sizes of local, temporal, and biogeographic processes on the density of rosette gallers, we found that time had the largest effect size, which was greater than *S. altissima* genotype (*P* < 0.001), nutrients (*P* < 0.001) and insects (*P* < 0.001) (Fig. 5). The effect sizes of genotype and latitude did not differ from each other (*P* = 0.98), but both had greater effect sizes than nutrients (*P* < 0.01) or insects (*P* < 0.01). Finally, the effect sizes of the nutrients and insects were not different (*P* = 0.18).

## Discussion

Our results indicate that the density and size of ecosystems engineered by rosette gallers are mediated by a combination of local processes, including host-plant genotype and the presence of other insects, inter-annual variation, and latitude. These results indicate that both biotic and abiotic environmental factors have the potential to mediate the community-level consequences of engineering insects by influencing the size of habitat available to secondary users.

The effects of host-plant genetic variation observed in this study are consistent with our prior work in this system (Crawford et al., 2007; Crutsinger et al., 2009). Moreover, they are in line with results from other systems that demonstrate variation in the abundance of engineering insects among different host-plant species (Marquis & Lill, 2010). While a growing number of studies have focused on the cascading effects of host-plant genetic variation (Whitham et al., 2006; Hersch-Green et al., 2011), whether the strength or direction of those effects remains consistent over space (Tack & Roslin, 2011) or time (e.g., Keith et al., 2010) remains an open question.

In this study, genetic variation in *S. altissima* influenced rosette galler density, but the effect varied from one year to the next. This suggests that susceptibility of genotypes might vary from one growing season to the next, perhaps depending on growing conditions for both gallers and host plants. Though we did not explore the specific role of weather in explaining temporal variation in gall density during this study, we did observe considerable inter-annual variability that could have influenced the quality, growth, and susceptibility of *S. altissima*. Similar to our results, Roche & Fritz (1997) measured resistance to 12 herbivore species across different half-sibs of silky willow (*Salix sericea*) for three years and found variation in resistance among genotypes was not consistent between years. In contrast, Keith et al. (2010) surveyed the total richness, abundance, and composition of a diverse arthropod community (103 species) on replicated clones of narrowleaf cottonwood (*Populus angustifolia*) for three years and observed no statistical interaction between host-plant genotype and time. Taken together, incorporating temporal variation in abiotic growing conditions into more studies will be critical to understanding the links between host-plant genetic variation and the diversity of associated communities. We suggest that other investigators, if they are not already, should examine whether and why genetic effects vary temporally, or are mediated by the environment (Keith et al., 2010; Breza et al., 2012).

We observed no response of rosette galler density to soil nutrient additions, which was surprising given that several studies have demonstrated that soil fertility can positively influence insect density through its influence on biomass and quality of host plants (Schade et al., 2003; Cornelissen & Stiling, 2006; Joern & Laws, 2013). Nutrient content can also exert a strong effect on insect herbivore populations by affecting their oviposition behavior, growth rates and population dynamics (Mattson, 1980; Denno et al., 2005; Huberty & Denno, 2006; Joern & Laws, 2013). However, the effect of increased plant quality on herbivores varies within and between insect species and feeding guilds (Awmack & Leather, 2002). Galling insects may be less responsive to the nutrient quality of their host plants because they create nutrient sinks on the plants to their own benefit (Stone & Schönrogge, 2003; Cornelissen & Stiling, 2006; Inbar et al., 2009). We did observe a response of gall size to nutrient addition, with smaller gall sizes in carbon-added (i.e., nutrient reduced) plots. Prior work in this system documented a positive relationship between the number of larval chambers and gall size, which suggests that rosette gallers lay fewer egg chambers in carbon-added plots, thereby creating smaller galls (Crutsinger et al., 2009).

We found that rosette gall density increased in plots where insects were removed using insecticide. Though it is unclear why rosette gallers were more abundant in these plots, there are possible explanations for this pattern. First, rosette galling adults could somehow be attracted to the pyrethroid-based insecticide. More likely, however, rosette gallers prefer plots where the abundance of potentially competing herbivores is reduced. Such a mechanism would be consistent with patterns of other galling species on *S. altissima* where there was greater host preference and larval performance on plants free of other insect herbivores, possibly because of the induction of plant defenses that would reduce host-plant quality (Cronin & Abrahamson, 1999; Cronin & Abrahamson, 2001; Helms et al., 2013). Rosette gallers might be avoiding competition with other herbivorous species (Prior & Hellmann, 2010), despite historical opinions that competition between phytophagous insects is inconsequential (Kaplan & Denno, 2007). An additional explanation for increased rosette gall density is that insecticide reduced insect predators or parasitoids of rosette gallers. As we did not measure parasitism rates of rosette gallers in the experiment, more research is needed to distinguish the underlying mechanisms driving higher gall abundances when insects are removed.

We observed an overall effect of time: gall densities in 2007 and 2008 were lower than 2004, 2005 and 2006. We also found a significant interactive effect of insects × year on rosette gall density (Table 1). This interaction was driven by 2004 when spring gall initiation could potentially have occurred prior to the establishment of the experimental treatments. The fact that overall densities of galls showed concurrent temporal patterns across the local experiments at the same study site (Freels Bend) adds further support to the notion that inter-annual variation is an important driver of engineering insect populations. There are few comparable studies exploring temporal variation in engineering insects. Sigmon & Lill (2013) explored leaf-tying caterpillars and associated arthropod community within ties on oak and birch trees throughout a growing season. They found that caterpillar and arthropod density per tie increased on both tree species as the season progressed, suggesting the intra-annual variation may also be important to consider in the study of engineering insects.

While it’s clear that local biotic and abiotic interactions can influence rosette gall density, we also observed large-scale variation across latitude, with higher mid-latitude densities. These patterns could be due to differences in underlying climatic factors, such as temperature and precipitation (Hopp & Foley, 2001; Rand & Louda, 2006; Fergnani et al., 2008) or local processes, such as competition or predation (Kaplan & Denno, 2007). Intraspecific patterns of herbivore abundance across latitudinal gradients are not well studied, but are becoming increasingly more common. In some species, abundance either decreases, peaks at mid-latitudes, or has no relationship with latitude (Andrew & Hughes, 2005; Pennings et al., 2009). The hump-shaped pattern might be explained by the ‘Abundance-centered hypothesis’ which posits that the abundance of a species is highest under the most favorable biotic and abiotic conditions, which are expected to occur at the center of its range, gradually decreasing toward the margins (Brown, 1984; Holt & Keitt, 2005). We could find little data on the range of *R. solidaginis*, though rosettes were present in all our most southern sites in Florida to the second most northern site in Vermont. We did not replicate the latitudinal transect across multiple years, and so are unable to directly compare the effect sizes of biogeography and time. It is likely that temporal variation has considerable effects on rosette gall density at biogeographic scales, as it did in our local experiments. Moreover, study sites were chosen haphazardly throughout the latitudinal range of *S. altissima*. Replicated common gardens distributed across the range might yield stronger latitudinal effect sizes than those in our observational study.

## Conclusions

Our results suggest that the density of rosette gallers is influenced by processes operating across a range of spatial and temporal scales, from local to biogeographic. Yet, when we compared the strength of these effects, we found the influence of time and host-plant genetic variation were the strongest predictors of rosette gall density. Although plant genetic variation has been shown to have strong effects on associated arthropod communities (Crutsinger et al., 2006; Whitham et al., 2006; Whitham et al., 2012), we are only beginning to understand the relative importance of genetic variation relative to other biotic and abiotic factors, or how those other environmental factors might mediate the effects of host-plant genotypes on associated communities (Hughes et al., 2008; Hersch-Green et al., 2011). While this study compared local and biogeographic factors independently, a further challenge lies in replicating local experiments across space and time to examine the context dependency of the suite of factors that shape the distribution, diversity, and abundance of associated organisms, and especially of ecosystem engineers.
